# Novel *SLC19A3* Promoter Deletion and Allelic Silencing in Biotin-Thiamine-Responsive Basal Ganglia Encephalopathy

**DOI:** 10.1371/journal.pone.0149055

**Published:** 2016-02-10

**Authors:** Irene Flønes, Paweł Sztromwasser, Kristoffer Haugarvoll, Christian Dölle, Maria Lykouri, Thomas Schwarzlmüller, Inge Jonassen, Hrvoje Miletic, Stefan Johansson, Per M. Knappskog, Laurence A. Bindoff, Charalampos Tzoulis

**Affiliations:** 1 Department of Neurology, Haukeland University Hospital, Bergen, Norway; 2 Department of Clinical Medicine, University of Bergen, Bergen, Norway; 3 Department of Clinical Science, University of Bergen, Bergen, Norway; 4 Center for Medical Genetics and Molecular Medicine, Haukeland University Hospital, Bergen, Norway; 5 Computational Biology Unit, Department of Informatics, University of Bergen, Bergen, Norway; 6 K.G. Jebsen Centre for Research on Neuropsychiatric Disorders, University of Bergen, Bergen, Norway; 7 Department of Radiology, Haukeland University Hospital, Bergen, Norway; 8 Department of Pathology, Haukeland University Hospital, Bergen, Norway; 9 Department of Biomedicine, University of Bergen, Bergen, Norway; 10 KG Jebsen Brain Tumor Research Center, University of Bergen, Bergen, Norway; Innsbruck Medical University, AUSTRIA

## Abstract

**Background:**

Biotin-thiamine responsive basal ganglia disease is a severe, but potentially treatable disorder caused by mutations in the *SLC19A3* gene. Although the disease is inherited in an autosomal recessive manner, patients with typical phenotypes carrying single heterozygous mutations have been reported. This makes the diagnosis uncertain and may delay treatment.

**Methods and Results:**

In two siblings with early-onset encephalopathy dystonia and epilepsy, whole-exome sequencing revealed a novel single heterozygous *SLC19A3* mutation (c.337T>C). Although Sanger-sequencing and copy-number analysis revealed no other aberrations, RNA-sequencing in brain tissue suggested the second allele was silenced. Whole-genome sequencing resolved the genetic defect by revealing a novel 45,049 bp deletion in the 5’-UTR region of the gene abolishing the promoter. High dose thiamine and biotin therapy was started in the surviving sibling who remains stable. In another patient two novel compound heterozygous *SLC19A3* mutations were found. He improved substantially on thiamine and biotin therapy.

**Conclusions:**

We show that large genomic deletions occur in the regulatory region of *SLC19A3* and should be considered in genetic testing. Moreover, our study highlights the power of whole-genome sequencing as a diagnostic tool for rare genetic disorders across a wide spectrum of mutations including non-coding large genomic rearrangements.

## Introduction

Biotin-thiamine-responsive basal ganglia disease (BBGD) is an autosomal recessive disorder caused by mutations in the *SLC19A3* gene, encoding human thiamine transporter 2 [1]. The disease typically starts in early childhood or teens and is characterized by episodic encephalopathy, epileptic seizures and severe extrapyramidal dysfunction. MRI shows bilateral lesions of the striatum, cerebral cortex and brainstem. The course is invariably progressive and, without treatment, can lead to severe disability and death. Administration of high dose biotin and/or thiamine can improve clinical symptoms, although patient response has been variable [2–5]. A more aggressive form of the disorder starts in early-infancy, and causes rapidly progressive encephalopathy with epilepsy, lactic acidosis, psychomotor regression and early mortality. It remains unclear whether these patients respond to therapy [6]. A later-onset Wernicke-like syndrome has also been reported. This commonly starts in the second decade of life with epilepsy, ataxia, nystagmus and ophtalmoplegia and MRI shows lesions of the medial thalamus and periaqueductal grey matter, mimicking Wernicke’s encephalopathy. Thiamine supplementation ameliorates clinical dysfunction [7].

The mechanism by which *SLC19A3* mutations cause disease is only partly understood. The SLC19A3 protein functions as a transporter for thiamine (vitamin B1), which cannot be endogenously synthesized in humans and must be obtained from external sources. Although biotin has no known affinity for SLC19A3 [1] biotin supplementation leads to increased SLC19A3 expression, which may at least partially explain the observed clinical response [8].

In the brain, SLC19A3 protein is mainly localized at the basement membrane and perivascular pericytes of cerebral vessels and the choroid plexus, where it transports thiamine across the blood-brain barrier. SLC19A3 is also expressed in peripheral tissues, including the intestinal mucosa where it mediates thiamine absorption and renal tubules where it is involved in thiamine reabsorption to prevent loss through the urine.

SLC19A3 mutations are associated with low levels of free thiamine in the cerebrospinal fluid (CSF), but not peripheral blood of patients suggesting impaired transport into the central nervous system [9]. This is indeed in line with the selective neurological involvement seen in patients with *SLC19A3* mutations.

Thiamine plays a central role energy metabolism. Its phosphorylated form, thiamine pyrophosphate (TPP), is an essential cofactor for numerous enzymatic reactions including the function of three mitochondrial enzymes; the pyruvate dehydrogenase complex, α-ketoglutarate dehydrogenase involved in the citric acid cycle, and the branched-chain α-ketoacid dehydrogenase complex involved in branched amino acid degradation [5]. Thiamine deficiency impairs oxidative decarboxylation of pyruvate and α-ketoglutarate causing accumulation of pyruvate and lactate and failure of energy metabolism [10].

Definite diagnosis of BBGD requires the detection of pathogenic mutations in the *SLC19A3* gene. Low CSF levels of free thiamine can be used as a screening tool to select patients for genetic testing and may serve as a potential biomarker for treatment monitoring [9]. Although the disorder is inherited in an autosomal recessive manner, patients with single heterozygous mutations have been reported [8] suggesting possible compound heterozygosity with changes not readily detectable by conventional sequencing methods, such as rearrangements, deletions and mutations in regulatory elements affecting expression of the gene. Such mutations have not been reported, however, and the diagnosis of heterozygous patients remains uncertain.

Here, we report three patients from two families with BBGD caused by novel *SLC19A3* mutations. Moreover, we use whole-genome sequencing to elucidate the genetic aetiology of disease in two patients where only a single heterozygous pathogenic variant was identified by whole-exome sequencing.

## Patients and Methods

### Patients

Three patients from two families with similar clinical phenotypes were referred to our Unit of Neurogenetics, due to suspicion of hereditary neurological disease.

**Fig 1:**
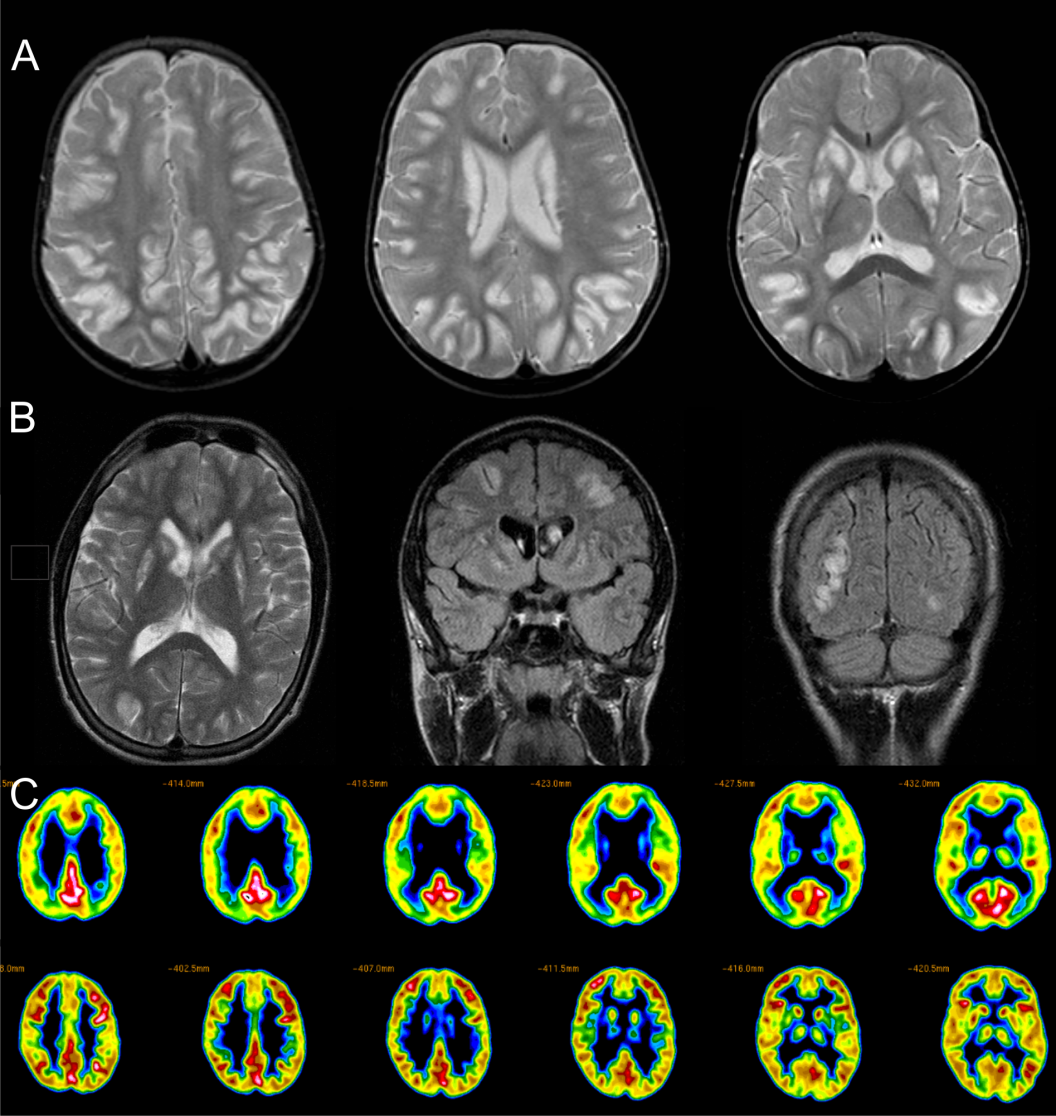
Neuroimaging findings. A: axial T2 weighted MRI of patient 2 taken during an episode with seizures, increasing encephalopathy and exacerbation of the dystonia. Images show bilateral high T2-signal changes and swelling in the putamen and caudate head. In addition there are multiple cortical lesions preferentially occurring in the depths of sulci, which is typical of BBGD. B: axial T2 and coronal T2-FLAIR weighted MRI of patient 1 taken during an episodic exacerbation show a similar pattern with bilateral striatal and multiple cortical lesions. The signal abnormalities regressed completely on later scans (not shown). C (upper lane): FDG-PET scan of the brain of patient 1 taken ~5 years later shows multiple foci of decreased glucose metabolism that correlate to the localization of the transient cortical signal changes on MRI. The striatum shows minimal uptake consistent with severe neuronal loss. A normal scan is shown in the lower lane for comparison.

Patients 1 and 2 were siblings born to non-consanguineous Norwegian parents. Both were born normally, after uncomplicated, full-term pregnancies and had normal early psychomotor development. The sister (patient-1) presented at the age of 13 months with psychomotor regression. Subsequently she developed an acute-on-chronic, progressive encephalopathy with generalized dystonia, epilepsy and episodic exacerbations with a combination of striatal and cortical lesions on MRI (Fig 1A). Clinical examination showed severe generalized bulbar, truncal and appendicular dystonia. She had no speech and severe dysphagia and was nourished through a percutaneous gastrostomy. Blood and urine tests were normal including complete metabolic screening. She died at the age of 15 years due to an acute exacerbation precipitated by pneumonia. Post-mortem examination was conducted.

The brother (patient-2) developed normally until the age of 12 months when he, too, developed a progressive encephalopathy with identical clinical features as his sister. Clinical examination at the age of 19 showed severe generalized dystonia affecting all muscle groups and causing aphonia and aphagia. Like his sister, he had to receive nourishment through a percutaneous gastrostomy.

Patient-3, a 24 year-old male, was born to non-consanguineous parents of Norwegian and Finnish ancestry. Pregnancy, birth and early psychomotor development were normal. At the age of thirteen months he developed generalized dystonia with a subacute-onset and chronic-progressive course. In addition he developed epilepsy with focal and generalized seizures and experienced several episodes with acute episodic hemiparesis followed by gradual recovery. Clinical examination at the age of 23 years showed generalized dystonia involving the bulbar muscles, trunk and limbs. He had a severe dysarthria and could walk short distances unsupported but with frequent falls. Routine biochemistry and metabolic screening were normal in blood and urine.

MRI of the brain performed during an episodic exacerbation at the age of 16 years showed bilateral lesions of the striatum and multiple cortical lesions affecting the depths of sulci as is typical for this disorder (Fig 1B)[11]. The cortical lesions had regressed on a subsequent MRI at the age of 18. FDG-PET scan of the brain at the same age however revealed multiple cortical hypometabolic foci which correlated to the transient MRI signal changes suggesting focal neuronal loss or persistent metabolic impairment (Fig 1C).

The index patients from both families (1 and 3) had earlier undergone extensive clinical, biochemical and conventional genetic investigations without findings. These included routine blood tests, serum levels and 24-hour urine excretion of copper and ceruloplasmin, routine CSF examination, full metabolic screening in blood and urine, autoimmune antibodies in serum and CSF. Sanger sequencing of *POLG*, *SUCLA2*, *DYT1*, and CAG-repeat assessment in *HTT* encoding Huntingtin revealed normal findings. Skeletal muscle biopsy was unremarkable by routine stainings as well as double histochemistry for cytochrome-oxidase and succinate dehydrogenase (COX/SDH). The full spectrum of mitochondrial DNA (mtDNA) changes was investigated in skeletal muscle including sequencing, quantification and deletion assessment and was unremarkable, excluding the possibility for primary mtDNA disease.

### Genetic Studies

The patients shared a similar clinical phenotype and family history was consistent with autosomal recessive inheritance. We therefore proceeded with whole exome sequencing (WES) of affected individuals, which is an established highly sensitive and cost-effective method for the diagnosis of recessive disorders [12–14].

DNA was purified from blood using standard methods. WES was performed on all three patients at HudsonAlpha Institute for Biotechnology (Huntsville, AL) using Roche-NimbleGen Sequence Capture EZ Exome v3 kit and paired-end 100nt sequencing on the Illumina HiSeq. The reads were mapped to human genome v37 using BWA aligner v6.2 [15], PCR duplicates removed with Picard v1.118 (http://broadinstitute.github.io/picard), and the alignment refined using Genome Analysis Toolkit (GATK) v3.2–2 [16] applying base quality score recalibration and realignment around indels as recommended in the GATK Best Practices workflow [17, 18]. Variants were called using GATK HaplotypeCaller [16] requiring a minimum coverage of 8 reads, and 5 reads for the variant allele. Filtering and annotation of variants was done in ANNOVAR [19]. Coding and putative splice sites were filtered against variants with MAF >0.8% in an in-house database of more than 300 Norwegian exomes, and variants present at >0.5% allele frequency in the 1000 Genomes database [20].

PCR-amplification and Sanger sequencing of the *SLC19A3* gene was performed by standard methods in all coding exons (2–6) and in addition 1,500 bases flanking each of the 5’ UTR and 3’UTR regions (primers and conditions available upon request). Copy-number variation (CNV) analysis was performed in patient-2 by quantitative PCR (qPCR) of each of the five coding exons of *SLC19A3* using standard methods (Centogene AG, Rostock, Germany).

RNA was purified from blood and brain tissue of patient-1 and three age-matched controls using commercial kits (Qiagen) and cDNA was synthesised using the SuperScript VILO cDNA Synthesis Kit (Invitrogen). Expression profiles for the *SLC19A3* gene were assessed in blood and brain by quantitative RT-PCR using a custom-made TaqMan assay and standard assays for beta-actin and GAPDH as endogenous controls. The complete RNA transcript was sequenced using standard methods (primers and conditions available upon request).

WGS was performed in patient-2 at HudsonAlpha Institute for Biotechnology (Huntsville, AL) using paired-end 100nt sequencing on the Illumina HiSeq. The reads were aligned to human genome v37 using BWA v6.2 [15], and data analysis was done in Integrative Genomics Viewer (IGV) [21]. The 45,049 bp deletion identified by genome sequencing was verified by specific PCR amplification of the break-points of the rearrangements (primer sequences available upon request).

### Western Blot Analysis

Frontal cortex tissue samples were homogenized in lysis buffer (50 mM Tris-HCl pH 8.0, 150 mM NaCl, 1 mM EDTA, 2% SDS). Protein concentration was determined by BCA protein kit (Pierce). SDS-PAGE and immunoblotting analyses were performed according to standard procedures. Primary rabbit anti-SLC19A3 antibodies were from Proteintech (13407-1-AP) and Sigma-Aldrich (SAB2103795) and mouse anti-GAPDH antibody were from Santa Cruz (sc-32233). HRP-conjugated rabbit anti-mouse (P0260) and swine anti rabbit (P0217) antibodies were from DACO. Enhanced chemiluminescence (SuperSignal, Pierce) was used for immunodetection. Images were taken using the ChemiDoc imager (Bio-Rad).

## Results

### Patients 1 and 2

WES in the two siblings revealed shared rare variants consistent with autosomal recessive inheritance in 7 genes, none of which had a known link to the phenotype. Both siblings were however heterozygous for the c.337T>C, p.Y113H mutation in the *SLC19A3* gene. Sanger sequencing of all *SLC19A3* exons (1–6) and 1,500 bases flanking each of the 5’ UTR and 3’UTR regions confirmed the p.Y113H mutation, but detected no other variants of pathogenic potential. The p.Y113H was absent in 400 in house controls and the 1000 genomes database [20]. It had an allele frequency of 5.77 x 10^−5^ (no homozygous individuals) at the Exome Aggregation Consortium (ExAC), Cambridge, MA (URL: http://exac.broadinstitute.org). Due to the likely deleteriousness of the p.Y113H mutation and compatible phenotype, we proceeded to examine the possibility of large-scale re-arrangements or regulatory mutations affecting expression of the wild-type *SLC19A3* allele.

WES coverage of the *SLC19A3* gene was optimal in the coding exons 2–6 (mean 83x and 76x for patients 1 and 2 respectively) but showed no evidence of copy-number changes. The non-coding exon-1 was not targeted by the exome capture assay.

SLC19A3 mRNA expression was undetectable in blood of both patients and controls consistent with the reported low expression in that tissue [22]. In brain tissue from patient-1, RNA sequencing revealed only a transcript from the allele carrying the c.337T>C mutation, suggesting that the second allele was not expressed.

**Fig 2:**
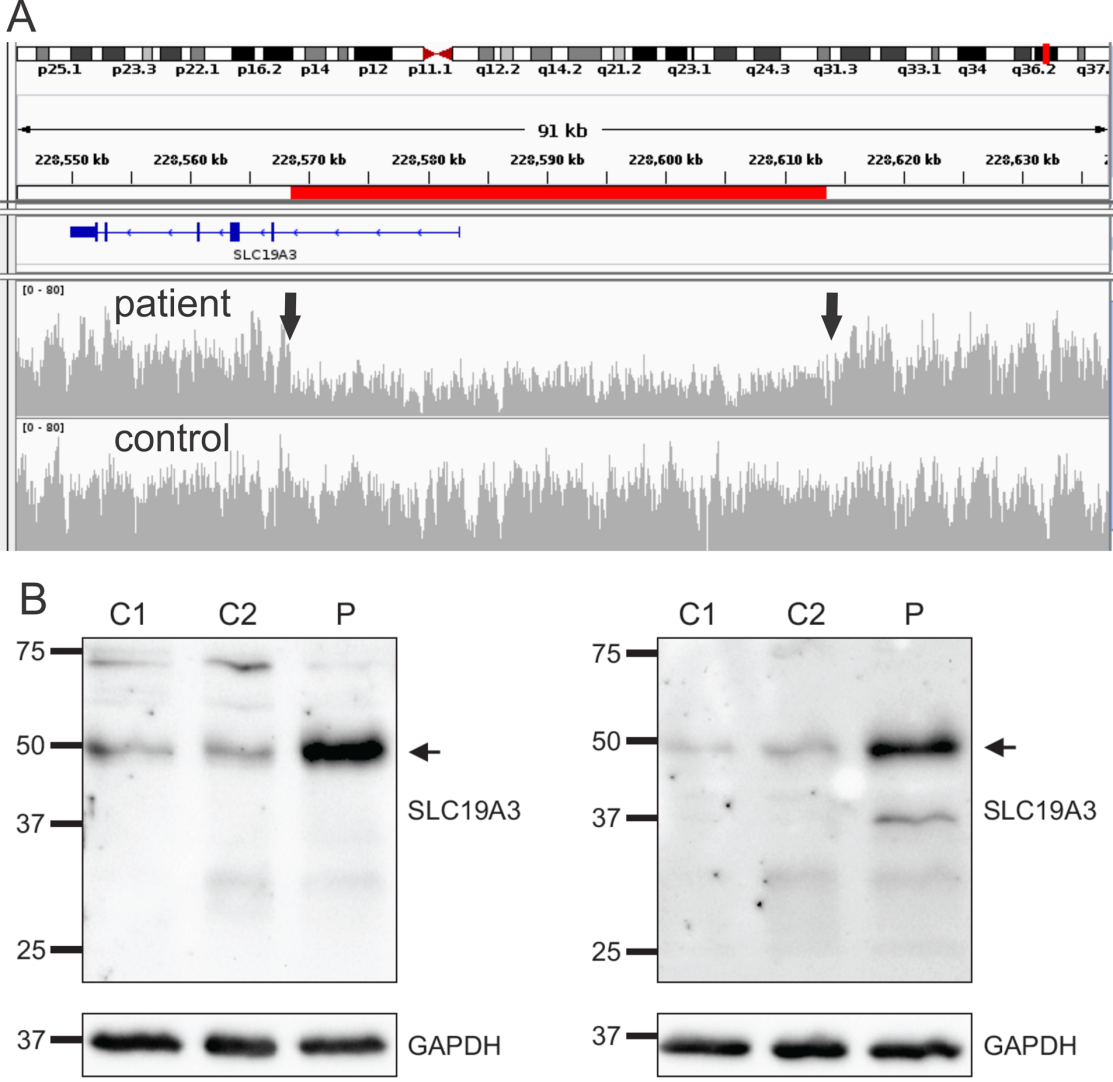
Genetic and molecular findings. A: coverage analysis of whole-genome sequencing data in patient 3 shows a clear regional drop in sequencing depth (red line) corresponding to the 45,049 bp deletion in the 5’-UTR of the *SLC19A3* gene. The two arrows mark the position of the break-points. Coverage in the same region of a control is shown in the bottom lane for comparison. The blue diagram depicts the SLC19A3 gene in a 3’-5’ alignment. Vertical rectangulars show the proportional position of the exons. B: Western blot analysis in frontal cortex homogenate of patient 1 using two primary antibodies (left and right panel) shows an increase of SLC19A3 protein immunoreactivity in the patient (P) compared to two age and gender matched controls (C1, C2).

CNV analysis by qPCR of all coding exons did not detect any deletion/duplication in the gene. In order to detect large-scale rearrangements and non-coding mutations we performed WGS of patient-2. Results showed a large deletion of 45,049 bp at chr2: 228,568,440–228,613,489 (Fig 2A). Targeted PCR amplification of the region spanning the deletion break-points confirmed that both siblings were heterozygous for the deletion. Analysis of the mother identified only the 45 kb deletion confirming that the two mutations are localized *in trans*.

Next we sought to determine the effects of the mutation on the protein level by Western blot in the patient´s brain tissue. Total SLC19A3 protein levels were significantly increased in the frontal cortex of patient-1. Western blot analysis in brain tissue using two distinct primary antibodies showed increased SLC19A3 immunoreactivity around 50kD (theoretical Mr ~56kD) in patient-1 compared to two controls (Fig 2B).

Patient-2 was given oral thiamine and biotin (300 mg b.i.d) with no apparent clinical effect during the first 3 months. The dose was subsequently doubled and he is currently clinically stable, but with no definite clinical signs of improvement at 6-month follow-up. This unresponsiveness to treatment has been described previously [5], and is probably a result of long lasting disease with subsequent permanent damage to the affected areas in the brain.

### Patient 3

WES revealed two very rare heterozygous mutations in exon-3 of *SLC19A3*: c.337T>C, p.Y113H and c.541T>C, p.S181P. Sanger sequencing confirmed the mutations. Visual inspection of WES data revealed that the heterozygous mutations were located on different strands (all pair-end reads spanning both positions contained only one of the mutations). The p.Y113H is described above for patients 1 and 2. The p.S181P was absent in 400 in-house controls and the 1000 genomes database [20] and had an allele frequency of 9.06 x 10^−5^ (no homozygous individuals) in the ExAC database. Both residues are moderately conserved.

Due to a compatible phenotype and very low minor allele frequency (MAF), the mutations were considered pathogenic and the patient diagnosed with BBGD. He was started on oral high dose thiamine (300 mg b.i.d) and biotin (300 mg b.i.d) with a substantially positive response in the form of clear improvement of the dysarthria and dystonia. Six months after treatment start he could walk unaided for long distances without falls and had begun to play soccer.

## Discussion

Using WGS, we resolved the cause of disease in a family with severe progressive encephalopathy and an apparently single heterozygous mutation in *SLC19A3*. We discovered a novel, large genomic deletion in the promoter region of the *SLC19A3* silencing the affected allele. We also report two missense mutations giving the substitutions: p.Y113H and p.S181P. These are predicted to be pathogenic due to very low population frequencies and segregation with a highly consistent phenotype.

The ~45 kb deletion in our patients affects the entire 5’-UTR region including the minimal promoter region required for basal expression [23]. RNA sequencing studies in brain tissue confirm that the deletion completely abolishes expression of the affected allele. Interestingly, total SLC19A3 protein levels were increased compared to controls suggesting a possible compensatory increase in the expression of the non-deleted allele carrying the c.T337C mutation. This observation is in agreement with the finding of increased SLC19A3 protein levels in the cerebral vessels of patients with *SLC19A3* mutations [11].

Non-coding deletions have not been reported in BBGD although their existence has been suggested in mutation negative cases with a typical phenotype but only a single heterozygous variant [8]. Such mutations may escape detection by conventional methods. The large size of the deletion in our patients and its location in the 5’-UTR region of the *SLC19A3* gene prevented its detection by Sanger sequencing or WES. Copy number analyses such as qPCR and multiplex ligation-dependent probe amplification (MLPA) would be sensitive for this type of mutation, but routinely done assays are commonly limited to the coding exons of the gene, which is why the deletion was initially missed in our case.

Early and sensitive diagnosis of treatable neurogenetic disease is crucial in order to prevent long-term complications and disability. BBGD is rapidly progressive with an episodic course and can lead to severe disability and death. Even though episodes are transient and cortical lesions may appear to regress by structural imaging, functional investigations with FDG-PET suggest that widespread neuronal loss may be inflicted with permanent consequences.

Another potential explanation for the PET findings is low cerebral glucose uptake due to lack of thiamine, which is a cofactor of the pyruvate dehydrogenase complex.

Our study highlights the power of WGS as a diagnostic tool for rare genetic disorders across a wide spectrum of mutations including non-coding large genomic rearrangements. We show that genomic deletions involving the regulatory region of *SLC19A3* should be considered in patients with compatible phenotypes where sequencing and coding CNV analyses are normal or only reveal a single heterozygous mutation. Such rearrangements can be detected by targeted CNV assays of the promoter area. A two-step approach combining conventional sequencing and CNV-analyses may however be a time-consuming and costly process. Given the increasingly effective analysis pipelines and rapidly declining cost, a direct application of WGS may soon be a more informative, cost- and time-efficient method for diagnosing genetic disease associated with a wide spectrum of genomic aberrations.

## Ethical Considerations

Our study was approved by the Regional Committee for Medical and Health Research Ethics, Western Norway (REK 2010/153). All study participants provided written informed consent.

## Abbreviations

BBGD: biotin-thiamine responsive basal ganglia disease
CNV: copy-number variation
FDG-PET: fluorodeoxyglucose positron-emission tomography
MRI: magnetic resonance imaging
WES: whole-exome sequencing
WGS: whole-genome sequencing
